# Draft Whole-Genome Sequences of Xylella fastidiosa subsp. fastidiosa Strains TPD3 and TPD4, Isolated from Grapevines in Hou-li, Taiwan

**DOI:** 10.1128/MRA.00835-19

**Published:** 2019-11-21

**Authors:** Andreina I. Castillo, Shu-Jen Tuan, Adam C. Retchless, Fei-Ting Hu, Hsun-Yin Chang, Rodrigo P. P. Almeida

**Affiliations:** a Department of Environmental Science, Policy and Management, University of California, Berkeley, Berkeley, California, USA; b Department of Entomology, National Chung Hsing University, Taichung, Taiwan, Republic of China; Portland State University

## Abstract

We report the draft assemblies of TPD3 and TPD4, two Xylella fastidiosa subsp. fastidiosa isolates infecting grapevines in Hou-li, Taiwan. TPD3 and TPD4 showed similar characteristics regarding genome size (2,483,503 bp and 2,491,539 bp, respectively), GC content (51.49% and 51.47%, respectively), and number of protein-coding sequences (2,394 and 2,413, respectively).

## ANNOUNCEMENT

Xylella fastidiosa is a xylem-limited plant-pathogenic bacterium that causes disease in crops and in ornamental and shade tree species (1). X. fastidiosa is transmitted by xylem sap-feeding insects of the Cicadellidae (sharpshooter leafhopper) and Cercopoidea (spittlebugs) families (2, 3). Disease symptoms associated with X. fastidiosa infection in grapevines were first described in Los Angeles, CA, by Newton Pierce in 1880 and have been subsequently referred to as Pierce’s disease (PD) of grapevine (4). The characteristic symptoms of PD include leaf scorching, gradual leaf chlorosis, and shriveling of grapes. Each year, X. fastidiosa infections cost $56.1 million in production losses, and $48.3 million in prevention costs are taken by nurseries and government agencies in the state of California (5). The symptoms described in California crops have also been reported on grapevines (Vitis vinifera L.) in central Taiwan in 2002 (3, 6). Sequencing of isolates obtained from infected grapevines in Taiwan showed that they share identical 16S rRNA sequences with X. fastidiosa PD strains from the Americas and are distantly related to Xylella taiwanensis (7). The results suggested that X. fastidiosa was imported to Asia from the Americas. Likewise, there have been multiple introductions of X. fastidiosa from the Americas to Europe (8, 9), as well as introductions of different subspecies within the American continent (10, 11). The expanding distribution and host range of X. fastidiosa bring forward relevant questions, mainly, what factors drive successful host infection and induction of symptoms in certain plant types, and how can better control, detection, and management strategies be developed? Since these factors have a genotypic component, whole-genome sequencing and subsequent analyses are expected to be helpful tools in answering these questions.

Two isolates were obtained from symptomatic grapevines in Hou-li, Taiwan (TPD3, 24°18′57.40″N, 120°41′53.30″E, and TPD4, 24°19′52.40″N, 120°42′03.90″E) in 2012. Petioles from symptomatic plants were wiped with 70% ethanol, sterilized by 0.6% NaOCl, and finally rinsed with sterile reverse-osmosis water (3). The sterile petioles were minced in 1 ml of PD2 broth (containing the following in g/liter: tryptone [4.0], soytone or phytone [2.0], trisodium citrate [1.0], disodium succinate [1.0], hemin chloride [0.01], MgSO_4_·7H_2_O [1.0], KH_2_PO_4_ [1.0], K_2_HPO_4_ [1.5], Bacto-agar [15.0], and bovine serum albumin fraction five [2.0]) using a razor blade in a petri dish. Samples were then grown in periwinkle wilt modified (PWG; containing phytone peptone [4.0 g], Trypticase peptone [1.0 g], KzHPO_4_, [1.2 g], hemin chloride stock [0.1% bovine heroin chloride in 0.05 N NaOH, 10 ml], KH_2_PO_4_ [1.0 g], GELRITE [9 g], MgSO_4_·7H_2_O, [0.4 g], phenol red stock [0.2% phenol red in distilled water, 10 ml], glutamine stock [8.0% {wt/vol}, 50 ml], and bovine serum albumin fraction-five stock [20% {wt/vol}, 15 ml]) medium plates, and isolation of a pure clone was done using a streak plate technique done three times in consecutive petri dishes. Subsequently, DNA was extracted using the Qiagen DNeasy blood and tissue kit (spin columns) using the Gram-negative bacterial DNA extraction protocol. The DNA provided for Illumina library preparation was at a concentration of 10.06 ng/μl and an *A*_260_/*A*_280_ purity ratio of 1.55 for TPD3. In the case of TPD4, the DNA concentration was 9.67 ng/μl, and the *A*_260_/*A*_280_ purity ratio was 1.42. Fragments were selected to be 400 bp for both TPD3 and TPD4. Isolates were sequenced using an Illumina HiSeq 2000 platform at the University of California, Berkeley Vincent J. Coates Genomics Sequencing Laboratory (California Institute for Quantitative Biosciences [QB3]). The quality of raw FASTQ paired reads was evaluated using FastQC (12). Low-quality reads and adapter sequences were removed from all paired raw reads using seqtk v1.2 (13) and cutadapt v1.14 (14), respectively. Briefly, seqtk uses Mott’s algorithm to perform quality trimming. The algorithm is described online at http://www.phrap.org/phredphrap/phred.html. In addition, cutadapt searches for provided adapter sequences (detected by FastQC) in all reads and removes them when it finds them. A total of 9,914,835 paired fastq reads were used for the TPD3 assembly, and a total of 10,054,122 paired fastq reads were used for the TPD4 assembly. Preprocessed Illumina reads were assembled *de novo* with SPAdes v3.13 (15, 16) using the -careful parameter and -k of 21, 33, 55, and 77. The assembled contigs were reordered using Mauve’s contig mover function (17) and the Temecula1 assembly (GenBank accession number GCF_000007245) as a reference. The TPD3 genome (818× sequencing depth of coverage) had a GC content of 51.49%, with a genome length of 2,422,083 bp distributed among 377 contigs ranging in size from 203 bp to 94,897 bp, an *N*_50_ of 38,877 kb, and an *L*_50_ of 20 kb. On the other hand, the TPD4 genome (828× sequencing depth of coverage) had a GC content of 51.47%, with a genome length of 2,427,175 bp distributed among 393 contigs ranging in size from 200 bp to 86,168 bp, an *N*_50_ value of 31,881 kb, and an *L*_50_ of 27 kb. The assembled and reordered genomes were individually annotated using PGAP (18). The TPD3 genome was predicted to have a total of 2,394 coding sequences (CDS), 51 tRNAs, 3 rRNAs, and 1 transfer-messenger RNA (tmRNA). The TPD4 genome was predicted to have 2,413 CDS, 51 tRNAs, 3 rRNAs, and 1 tmRNA.

**FIG 1:**
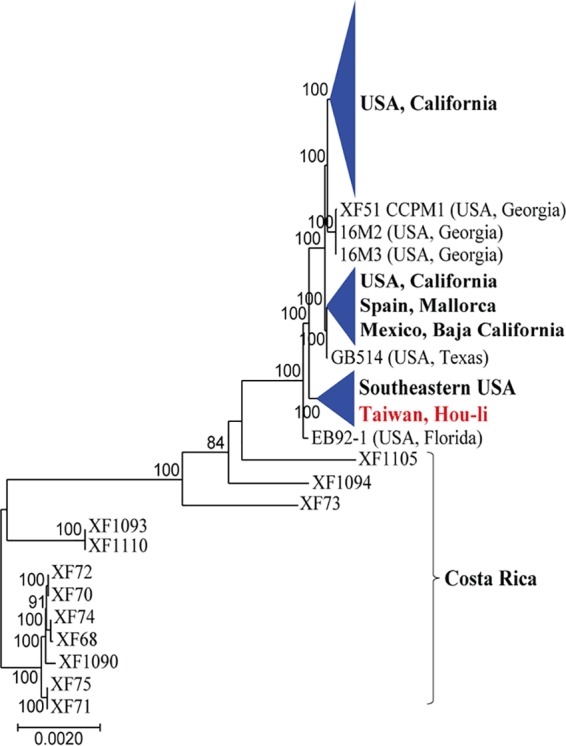
Phylogeny build using the core-genome alignment for worldwide X. fastidiosa subsp. fastidiosa isolates. Both samples from Hou-li, Taiwan (red), cluster within the clade, including samples from the southeastern United States, suggesting that they descend from this population.

Roary v3.11.2 was used to create an alignment of genes shared in 99% to 100% of the isolates in a data set (core-gene alignment) (19). The core-genome alignment was used to build a maximum likelihood (ML) tree using RaxML (20). The tree was built using the GTRCAT substitution model. Tree topology and branch support were assessed using 1,000 bootstrap replicates. The core-genome phylogenetic analyses of isolates TPD3 and TPD4 show a clear clustering within other X. fastidiosa subsp. fastidiosa isolates originating from the United States, thus providing further evidence for an introduction from the Americas into Taiwan (Fig. 1).

### Data availability.

All raw reads and information regarding each strain have been submitted under BioProject number PRJNA549761. TPD3 is submitted under BioSample number SAMN12097273, and TPD4 is submitted under BioSample number SAMN12097274. The accession numbers are VJWG00000000 (assembly accession number GCA_007845655) for TPD3 and VJWH00000000 (assembly accession number GCA_007845705) for TPD4.
